# Braille Block Detection via Multi-Objective Optimization from an Egocentric Viewpoint

**DOI:** 10.3390/s21082775

**Published:** 2021-04-14

**Authors:** Tsubasa Takano, Takumi Nakane, Takuya Akashi, Chao Zhang

**Affiliations:** 1 Department of Engineering, University of Fukui, Fukui 910-8507, Japan; t-takano@monju.fuis.u-fukui.ac.jp (T.T.); t-nakane@monju.fuis.u-fukui.ac.jp (T.N.); 2 Faculty of Engineering, Iwate University, Iwate 020-8550, Japan; akashi@iwate-u.ac.jp

**Keywords:** Braille block detection, egocentric vision, multi-objective optimization

## Abstract

In this paper, we propose a method to detect Braille blocks from an egocentric viewpoint, which is a key part of many walking support devices for visually impaired people. Our main contribution is to cast this task as a multi-objective optimization problem and exploits both the geometric and the appearance features for detection. Specifically, two objective functions were designed under an evolutionary optimization framework with a line pair modeled as an individual (i.e., solution). Both of the objectives follow the basic characteristics of the Braille blocks, which aim to clarify the boundaries and estimate the likelihood of the Braille block surface. Our proposed method was assessed by an originally collected and annotated dataset under real scenarios. Both quantitative and qualitative experimental results show that the proposed method can detect Braille blocks under various environments. We also provide a comprehensive comparison of the detection performance with respect to different multi-objective optimization algorithms.

## 1. Introduction

**Figure 1:**
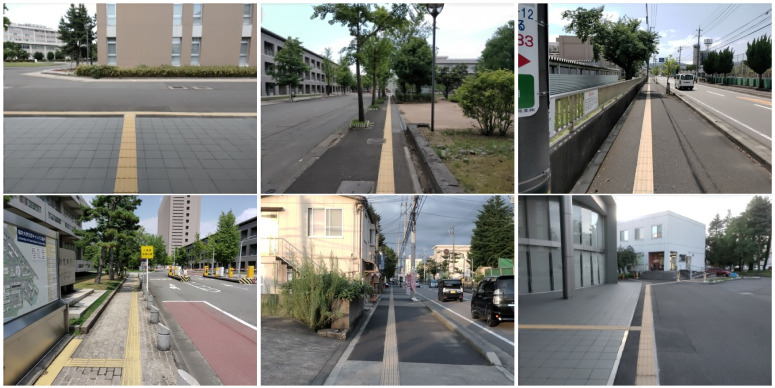
Examples of the Braille blocks from an egocentric viewpoint.

In the last decade, wearable devices have become widespread in a wide range of applications from healthcare to monitoring systems due to the development of miniaturization and computational power. Recent interests in navigation aid for blind people have spurred research aimed at the detection of obstacles and detecting the distance to nearby objects [1]. On the other hand, besides assistive techniques such as white canes and guide dogs, tactile paving (also known as Braille blocks or tenji blocks) is ubiquitous in Japan, which is a system of textured ground surfaces to assist pedestrians who are visually impaired (e.g., Figure 1). As one of the most important usages, the surface of Braille blocks is designed to be uneven such that people can be guided along the route by maintaining contact with a long white cane. However, cane travel can be cumbersome and not as fluid because of its weight and the physical effort required to swing. To eliminate the inconvenience brought by the Braille blocks, one possible solution is to develop a head-mounted device embedded with a sensory substitution system for cane-free walking support. As the first step, the device is required to automatically locate the region of Braille block in the image taken by the first-person camera, which is also the main purpose and motivation of this paper.

In real-world problems, there may exist multiple objectives to be optimized simultaneously in order to solve the task. Multi-objective optimization (MO) is a technique to solve such tasks with the results represented by the Pareto optimal solution, which is a set of non-dominated solutions. The Pareto optimal solution allows for compromises between different evaluation criteria, without favoring one over the other, and thus gives a reasonable solution considering the trade-off. In this paper, the MO technique can be applied to the problem of Braille block detection by assessing multiple types of features of Braille blocks in the form of calculating multiple objective functions. As the basic strategy, we consider that the task of Braille block detection can be effectively solved under an optimization framework due to the simple geometric and appearance features. Specifically, in this paper, the popular multi-objective genetic optimization algorithm, non-dominated sorting genetic algorithm-II (NSGA-II) [2], was used to optimize multiple validity measures simultaneously. The main contributions of this paper are threefold.

- A Braille block detection framework with the egocentric images as input is proposed.
- We formulate the block detection as a multi-objective optimization problem by considering both the geometric and the appearance features.
- A Braille block detection dataset is originally built with annotations.

The paper is organized as follows. In the next section, we introduce related work. Section 3, we present the proposed framework using MO. Section 4, we describe the qualitative and quantitative experimental results of Braille block detection in egocentric images. The conclusion is presented at the end of this paper.

## 2. Related Work

In the field of egocentric vision, object detection and recognition [3,4,5,6,7,8] is a popular problem. To the best of our knowledge, Braille block detection in the form of egocentric vision has been sparsely treated so far. Yoshida et al. [9] propose a strategy to recognize Braille blocks using a sensor to detect bumps on road surfaces in autonomous mobile robot navigation. This method requires a particular sensor that cannot be used for the detection of Braille blocks from images. Okamoto et al. [10] used a convolutional neural network that learned from more than 10,000 images of training data to detect Braille blocks in images. This method requires a large amount of computational and labor costs in training, collecting data and tuning parameters despite the fact that the pattern of Braille blocks is fairly simple. Therefore, instead of collecting large amounts of data to improve accuracy, we propose the extraction of the geometric feature (linearity) and the appearance feature (yellow color) of the Braille blocks. To measure the validity of each feature, two objectives were designed and the optimal solution was achieved under the MO framework.

On the other hand, geometric feature extraction (shape recognition) research using evolutionary algorithms (EA) has been studied for a long time. Ever since Roth et al. showed that geometric primitive extraction can be treated as an optimization problem and genetic algorithm (GA) can be applied to it [11,12], various methods using EA have been proposed. Generally, in these methods, a candidate shape for a solution (i.e., an individual) is represented as a combination of multiple points, and an objective function is designed to verify whether the solution candidates actually exist on the feature space or not. Chai et al. [13] proposed an optimization method called evolutionary tabu search (ETS), which is a combination of GA and tabu search (TS) algorithm, for geometric primitive extraction. The experimental results show the superiority of ETS in detecting ellipses from images and comparing it against optimization algorithms such as GA, simulated annealing and TS. Yao et al. [14,15] proposed Multi-Population GA, which optimizes a large number of subpopulations by evolving them in parallel, instead of evolving a single population as in the conventional GA, and showed its superiority compared to randomized Hough transform and shared GA in ellipse detection. Ayala et al. [16] proposed circle detection using GA. This method encodes an individual as a circle passing through three points and evaluates whether the circle actually exists in the edge image with an objective function. Their objective function evaluates the completeness of the candidate circle by assessing the percentage of pixels existing in the edge feature space. Değirmenci [17] showed that the parallelization capability of GPU can be used to extract geometric primitives using GA, resulting in a speedup compared to CPU. Raja and Ganesan [18] proposed a fast circle detection based on GA that reduces the search space by avoiding infeasible individual trials. Also, there are several works for line detection. Lutton and Martinez [19] proposed to use GA for geometric primitive (segment, rectangle, circle and eclipse) extraction from image. Their method uses a distance transformed image to compute the objective function. Using distance transformed images, the landscape of the objective function can be smoothed and the similarity between an individual and the original image can be measured. Mirmehdi et al. [20] presented line segment extraction method using GA. The algorithm computes a quality scale from the statistics of gray-level values in the boxes on either side of the line segment. Kahlouche et al. [21] presented a method of geometric primitive extraction using objective function that is the sum of the average intensity of the distance transform image and the number of edge pixels on the trace of the primitive. GA is the most commonly used algorithm for geometric primitive extraction [12,13,14,15,16,17,18,19,20,21]. Besides, techniques that combine the advantages of particle swarm optimization (PSO), GA, chaotic dynamics [22], bacterial foraging optimization [23] and artificial bee colony optimization [24] are also effective alternatives. Also, other meta-heuristic search algorithms are adopted for shape search such as differential evolution (DE) [25,26], adaptive population with reduced evaluations [27] and harmony search [28].

Real-world objects can also be detected by detecting geometric shapes. Many studies have been conducted using geometric feature extraction with EA for real-world problems [29,30,31,32]. Soetedjo et al. [30] proposed to detect circular traffic signs from images using GA based eclipse detection. Cuevas et al. [31] proposed to detect white blood cells from medical images using elliptic detection with DE algorithm. Alwan et al. [32] adopted GA-based primitive extraction in vectorizing paper drawings. To solve multi-objective optimization problems (MOP) with two or three objectives, many multi-objective evolutionary algorithms (MOEA) have been proposed, such as strength Pareto evolutionary Algorithm2 (SPEA2) [33], NSGA-II [2], indicator-based evolutionary algorithm (IBEA) [34], generalized differential evolution 3 (GDE3) [35], multi-objective evolutionary algorithm with decomposition (MOEA/D) [36], non-dominated sorting genetic algorithm-III (NSGA-III) [37], improved decomposition-based evolutionary algorithm (DBEA) [38], etc. MO algorithms are generally designed to solve problems that require optimizing multiple objectives, and have been applied in the field of computer vision [39]. For example, Bandyopadhyay et al. [40] proposed land cover classification in remote sensing images with NSGA-II. This approach solves the problem by simultaneously optimizing a number of fuzzy cluster viability indexes. Mukhopadhyay et al. [41] proposed a multi-objective genetic fuzzy clustering scheme utilizing the search capability of NSGA-II, and applied it to the segmentation of MRI brain images. Nakib et al. [42] proposed image thresholding method based on NSGA-II. This method argues that optimizing multiple segmentation criteria simultaneously improves the quality of the segmentation. Shanmugavadivu et al. [43] proposed multi-objective histogram equalization using PSO to achieve two major objectives of brightness preservation and contrast enhancement of images simultaneously. In addition, image segmentation using NSGA-II [44], MOEA/D [45], and watermarking algorithms using multi-objective ant colony algorithms [46] have been proposed. Among them, NSGA-II is a commonly used MO algorithm for real-world problems when the number of objectives is small.

## 3. Detection of Braille Block

### 3.1. Problem Setting and Overview

**Figure 2:**
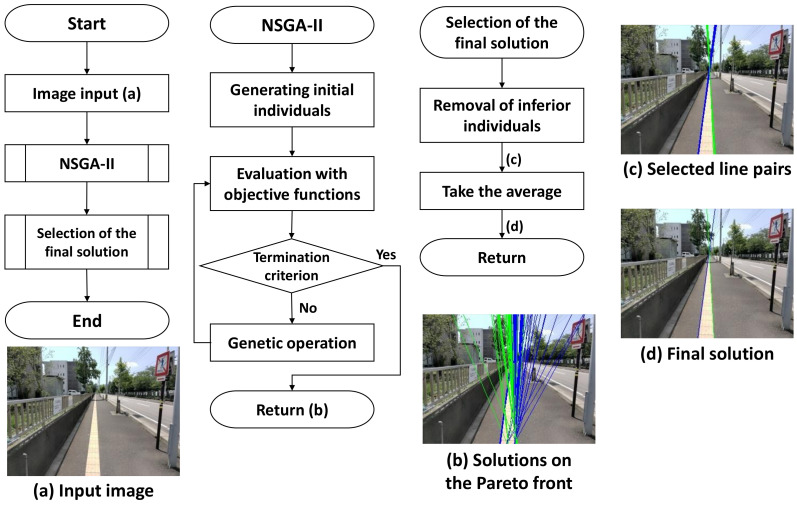
Overview of our proposed method.

The proposed method uses both appearance and shape features to extract the Braille block region from images taken by an egocentric camera mounted on a walking person. In our problem setting, we especially aim at extracting yellow Braille blocks with linear boundaries for preventing the blind people from straying from the route as shown in Figure 1. By assuming that the walking person is initially on the Braille block, we can observe from the images that the Braille blocks extending from the bottom to the top in a perspective view. Also, as the Braille blocks from the user’s egocentric viewpoint appear as regions bordered by two boundaries, the detection problem can then be treated as a task to locate yellow regions with a line pair as boundary lines. Detecting a line (segment) from an image can be understood as extracting a geometric primitive. The overview of our proposed MO based Braille block detection is shown in Figure 2. Each solution (i.e., individual) encodes parameters to define a pair of boundary lines. MO algorithm plays a role in finding solutions on a Pareto front to provide quality candidates considering both the geometric and color characteristics. After removing inferior individuals, the final solution is determined by averaging the survived individuals.

### 3.2. Individual Representation and Population Initialization

**Figure 3:**
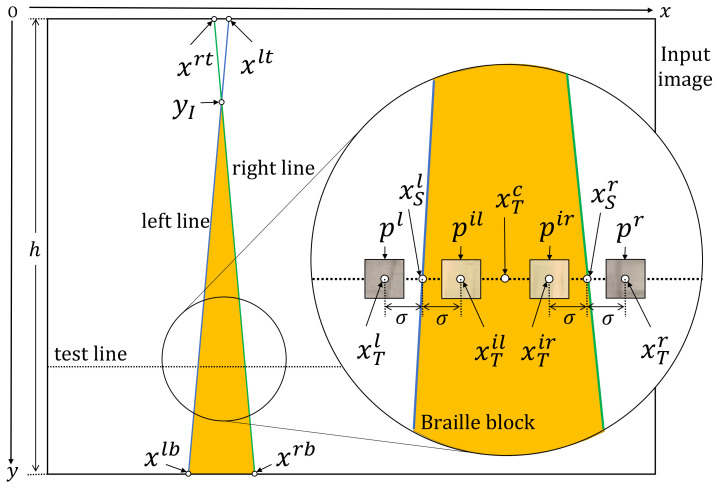
Notation of the variables used for calculating the objective functions.

We represent each individual with a pair of boundary lines, which implicitly defines the region of Braille blocks from an egocentric viewpoint. Each individual LP is real-coded, which means real variables are directly dealt with. In the perspective view, since the two boundary lines can be extended infinitely, and each extended line will have intersection points with the upper and lower boundaries of the image, respectively, only four *x*-axis coordinates $LP=\{x^{lt},x^{lb},x^{rt},x^{rb}\}$ are needed in total to define a line pair as shown in Figure 3. $x^{lt}$ and $x^{lb}$ represent the left-top and the left-bottom points of the left boundary line, and similarly, $x^{rt}$ and $x^{rb}$ represent right-top and the right-bottom points of the left boundary line. Each candidate solution LP in the initial population is randomly generated by sampling *x*-axis coordinates from the upper and the lower boundaries of the image. To accelerate the convergence and remove unpromising solutions in advance, individuals are initialized with limitations. That is, for each valid solution, the interval between the two lines at the image bottom is limited within [20,50] pixels, and the slope of the left line and the right line is limited to be smaller than 1/12π (clockwise and counterclockwise, respectively). Further, the *y*-coordinates of the intersection point are limited within [0,0.6*h*]. As the valid individuals are more likely to represent valid boundaries, such an initialization strategy is expected to contribute to reaching the optimal solution earlier and reducing false detection.

### 3.3. Objective Functions

In order to evaluate whether each LP represents a reasonable region of Braille blocks, two objective functions evaluating color and shape features are simultaneously optimized. In the ideal case (no complex background, occlusion or change in appearance), the two objective functions can work collaboratively to locate the Braille block region. However, under the real-world scenarios, as the Braille blocks will show various variations of appearance, rating the individuals in terms of the combination of two objectives will lead to bad solutions with either of the objective values being low (in this paper, the problem is cast as a minimization problem). That is, we aim to obtain a solution that satisfies both objectives to some extent while a solution that satisfies both objectives can hardly exist due to the interference under real-world scenarios.

**Figure 4:**
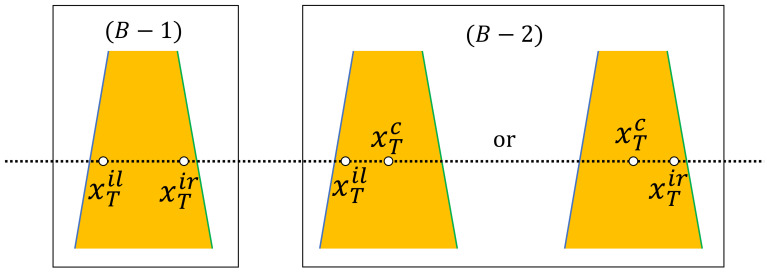
Test points in conditionB.

Figure 3 illustrates the variables used in the objective functions. In objective function 1, following the observation that Braille blocks are yellow, we use color histogram in the HSV color space to assess the following two facts: (1) the color histograms differ between the regions inside and outside the boundary lines; (2) the test pixels sampled from the region inside the boundary lines represent “yellow”. “Yellow” is predefined by an HSV range. The calculation process is summarized in Algorithm 1. Specifically, the HSV image of the input image is denoted by $I_{HSV}$. As illustrated in Figure 3, the four *x*-coordinates of the test points on the test line to assess the color histogram are denoted by $x_{T}=\left[x_{T}^{l},x_{T}^{il},x_{T}^{r},x_{T}^{il},x_{T}^{c}\right]$. With the test points as the centers, the test patches are denoted by $p=[p^{l},p^{il},p^{r},p^{ir}]$, and their color histograms are $hist=[hist^{l},hist^{il},hist^{r},hist^{ir}]$. In conclusion, $x_{T}$ is calculated from LP and the position of the test line, *p* depends on $x_{T}$ and $I_{HSV}$, and hist is calculated from *p*. The *y*-coordinate of the intersection point of the line pair is $y_{I}$. $y_{I}$ can be calculated from LP. If $y_{I}$ is on the image, then the test line takes the intersection point as the starting point and moves down pixel by pixel for dense tests because only the Braille block region needs to be tested, else the test line starts moving from the top of the image. Over all the test lines from the intersection point to the bottom of the image, we introduce two counters $c_{a}$ and $c_{b}$ to collect summary statistics that contribute to the fitness value with respect to different conditions. For condition(A): neither the similarity between $hist^{l}$ and $hist^{il}$ nor the similarity between $hist^{r}$ and $hist^{ir}$ is high. The purpose is to ensure clear boundary lines, which is intuitive. The similarity is calculated by comparing two histograms with respect to Bhattacharyya distance. For condition(B): HSV value of $x_{T}$ is within the predetermined range for defining “yellow”. The purpose is to ensure the existence of the Braille blocks. Furthermore, as shown in Figure 4, condition(B) has two sub-conditions for fine-grained tests in order to improve the robustness. For condition(B−1): $x_{T}^{il}$ and $x_{T}^{ir}$ are yellow. For condition(B−2), $x_{T}^{il}$ and $x_{T}^{c}$ are yellow or $x_{T}^{ir}$ and $x_{T}^{c}$ are yellow. Two counters are prepared and their weights are changed according to the test points, in order to improve the noise resistance. The center test point ($x_{T}^{c}$) is used as a remedy in case that $x_{T}^{il}$ and $x_{T}^{ir}$ are severely affected by noise. Counter $c_{a}$ only counts if the condition (B−1) is met, thus it contributes to position adjustment of the line pair, with low resistance against noise. $c_{b}$ counts when either condition (B−1) or (B−2) is met, thus a line pair can be fitted to the Braille block region allowing a certain level of noise. As the MO problem is set as a minimization optimization problem, the objective values $v_{1}$ is set to be negative.
**Algorithm 1:** Objective Function 1
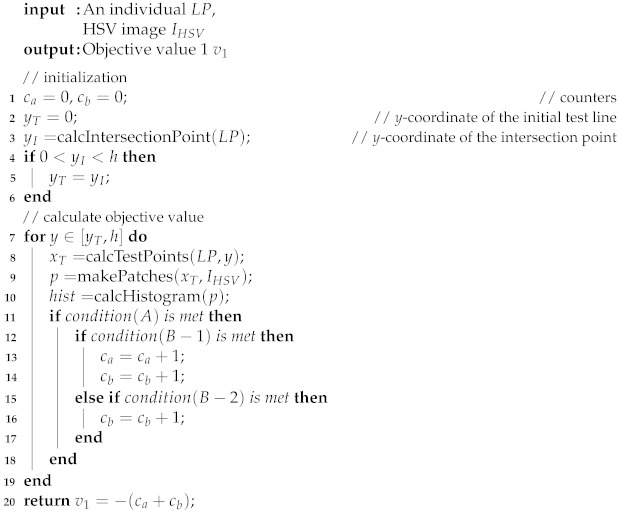



**Figure 5:**
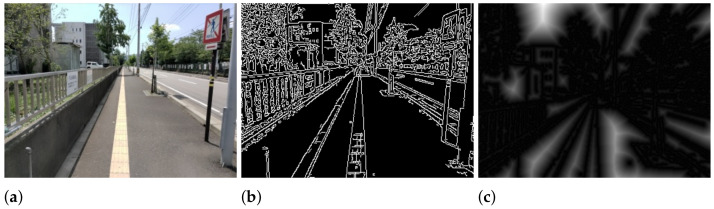
(**a**) Input image, (**b**) Edge image, (**c**) Distance transform image.

Objective function 2 exploits linear shape features as summarized in Algorithm 2. Specifically, given a distance transform image $I_{DT}$ transformed from the edge image, we aim to find the Braille block boundaries by minimizing the sum of distance. In $I_{DT}$, the value of each pixel $I_{DT}\left(x,y\right)$ is the Euclidean distance from the nearest edge as illustrated in Figure 5. Therefore, the sum of the pixel values of the points on the line pair below the intersection point can be treated as the likelihood of an individual representing boundaries. The *x*-coordinate of the sample points for calculating the distance are denoted by $x_{S}=\left[x_{S}^{l},x_{S}^{r}\right]$, which is illustrated in Figure 4.
**Algorithm 2:** Objective Function 2
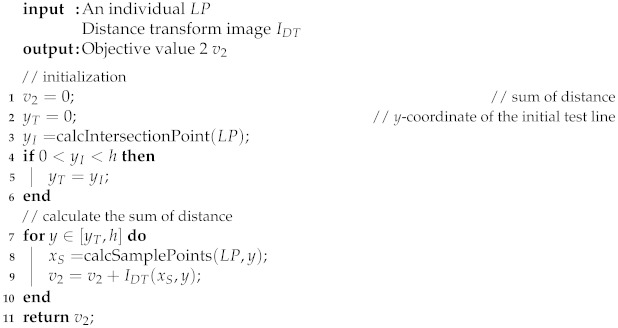


### 3.4. Genetic Operators and Termination Criterion

We adopt NSGA-II [2] as the main algorithm to solve our MO problem described in the previous section. Also, other popular MO algorithms are compared in Section 4. Specifically, three genetic operators including selection, crossover and mutation are used. Crowded binary tournament selection without replacement is used as the selection operator. To propagate the elite individuals found in the previous searches to the next generation, the non-dominant solutions for the parent populations are used based on the non-domination rank and crowding distance. For the crossover operator, the simulated binary crossover (SBX) operator [47] is used. SBX simulates a single-point crossover of binary-encoded real-valued decision variables. For the mutation operator, the polynomial mutation (PM) operator [48] is used. PM simulates binary-encoded bit-flip mutations in a real-valued decision variable. In our experiment, the crossover probability is taken as 1.0 as suggested by [49,50,51] and the mutation probability is set to 0.25. As to the termination condition, the iteration is run by a determined number of generations.

### 3.5. Selection of the Final Solution

To generate the final solution based on the solutions on the Pareto front in the last generation, we propose a two-stage strategy: first, remove the individuals that either the *y*-coordinate of the intersection point belongs to [0.6h,h] or overlapped, which turns out to be able to remove implausible solutions. Second, the average of the remaining individuals is taken as the final solution.

## 4. Experimental Results

The performance of our proposed method was evaluated by comparing the line pair result with the manually annotated ground truth over our originally collected dataset. Our dataset consists of 50 test images taken by the Vuzix M400 Smart Glasses, which includes five categories in total: illumination change, shadow, deficiency, obstacle and change of view angle. Each category contains 10 images in a size of 320 × 240 pixels. The MOEAs programs used in the experiment are obtained from MOEA Framework 2.13 (http://moeaframework.org/, accessed on 1 December 2020), a java open source library. Each numerical result is averaged by 10 trials with different random seeds. The parameters of NSGA-II and other MOEAs used in the experiments are shown in Table 1, and descriptions of the parameters used are listed in Table 2. For the quantitative evaluation, the evaluation criterion is set as the mean location error, which is calculated by the root mean square error (RMSE) defined as follows,

(1) $$RMSE={\left(\sum_{y=y_{G}}^{h}{\left(x_{R}-x_{G}\right)}^{2}/\left(h-y\right)\right)}^{1/2}.$$

where $x_{R}$ and $x_{G}$ are the *x*-coordinates of the final solution and the ground truth. $y_{G}$ is the *y*-coordinate of the intersection point of the ground truth.

### 4.1. Parameter Tuning

**Figure 6:**
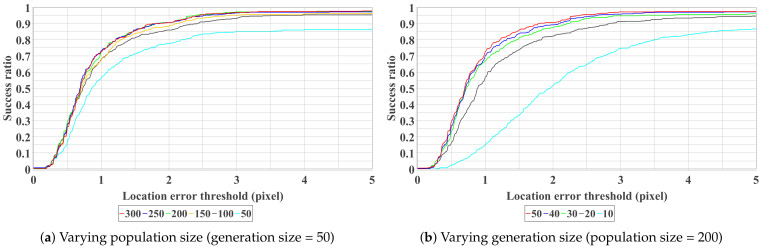
Curves show the average success ratio of 10 trials with different parameter settings. All the test images are used for plotting. The curve closer to the top left represents better performance. Best view in color.

First, the results by varying population size and number of generations are shown in Figure 6. It can be observed from Figure 6a that the results of population size = 200, 250 and 300 are very close, which indicates that it is difficult to improve the performance by increasing the population size after 200. On the other hand, with the population size fixed as 200, we can observe from Figure 6b that the number of generations is proportional to the performance and the improvement is trivial after 30. Finally, the optimum setting (population size = 200, generation size = 50) is adopted for the following experiments.

### 4.2. Performance Evaluation and Limitation Analysis

We present quantitative and qualitative results in this section. Figure 8a shows the overall accuracy with respect to the whole test dataset. As can be observed, a high success ratio (>0.9) can be achieved when the threshold value of the mean location error is larger than two pixels. Success ratio indicates the percentage of the test images that are successfully detected. The success ratio can further increase to 0.95 when three pixels of error are allowed for the detection result.

**Figure 7:**
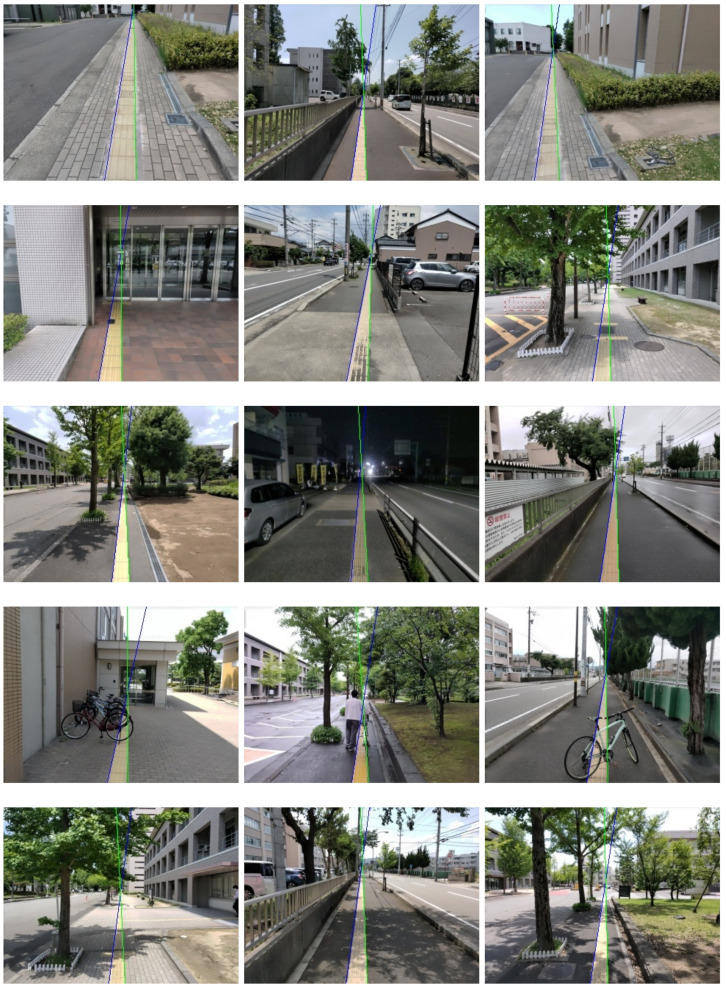
Category-wise qualitative results. A line pair consists of a blue line (**left**) and a green line (**right**). Categories from the **top** row to the **bottom** row: change of view angle, deficiency, illumination change, obstacle and shadow.

**Figure 8:**
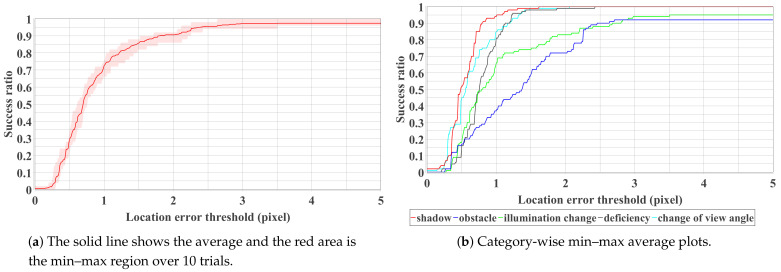
Success ratio plot with respect to the whole (**a**) and partial (**b**) dataset.

**Figure 9:**
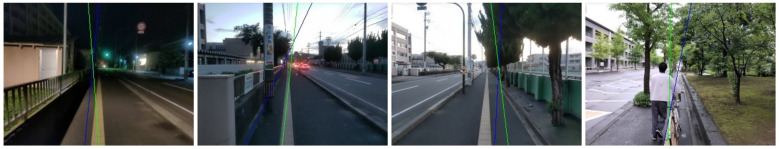
Examples of failure defections. **1st**∼**3rd** examples show the case of illumination change and the **last** example shows the case of obstacles.

Our proposed method can also detect Braille blocks under various environments as shown in Figure 7. From the quantitative analysis in Figure 8b, we can observe that our proposed method is especially robust to deal with shadows, deficiency and change of view angle. In the case of obstacles, as Braille blocks are partially covered by obstacles, color and shape features cannot be sufficiently obtained in some test images. In the case of illumination changes, color features are mainly affected, resulting in a decrease in the success ratio in both cases. False-positive detection is more likely to happen when either of the features is inadequate or one of the features is implausible. As can be observed in Figure 9, our proposed method has limitations especially when it gets dark or obstacles exist on the road. When the color range for defining “yellow” changes significantly, the predetermined HSV range becomes ineffective. Instead of a fixed range, an adaptive color range could probably solve this problem.

### 4.3. Comparison over Different MOEAs

**Figure 10:**
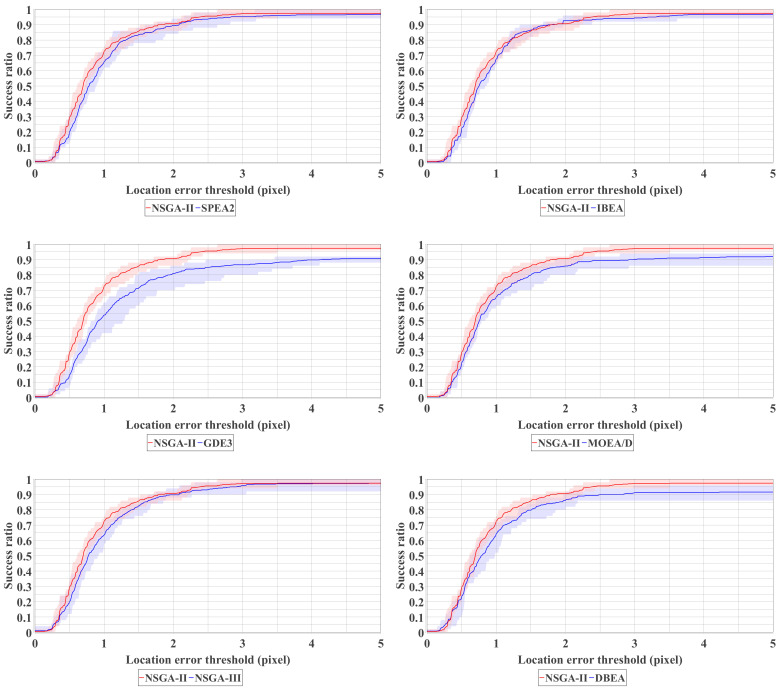
Comparative experiment. All test images are conducted 10 trials for the plots.

**Figure 11:**
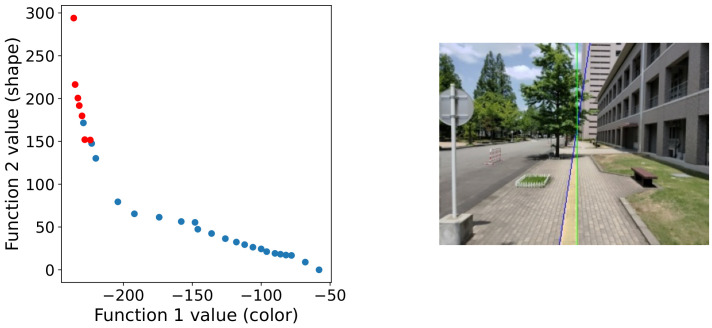

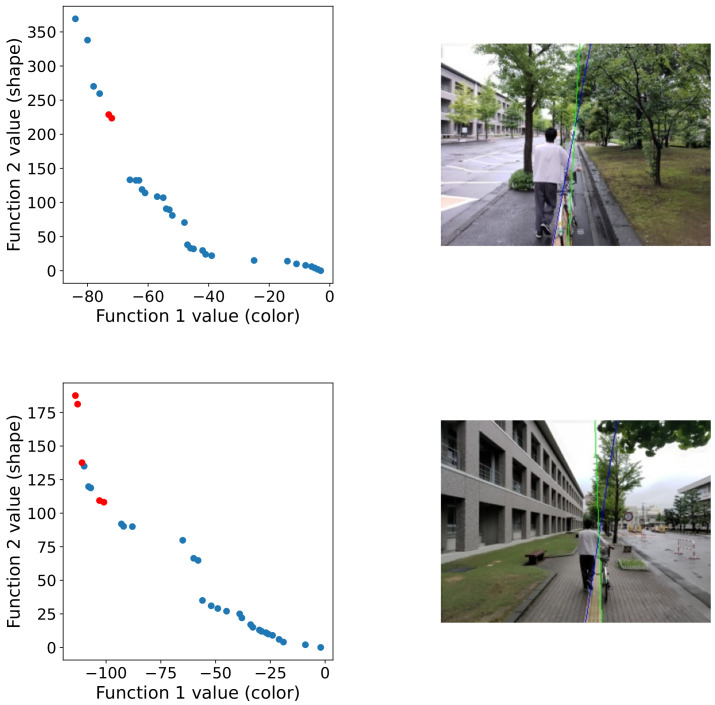
Pareto front approximation of NSGA-II in the final generation. The blue circles show the non-dominated solutions, and the red circles show the selected solution for final decision making (i.e., the average of the red circles is the final result plotted on the right image).

Despite NSGA-II, we also test other MOEAs for comparison and provide a reference for future studies. Six MOEAs for multi-objective optimization, namely, SPEA2 [33], IBEA [34], GDE3 [35], MOEA/D [36], NSGA-III [37] and DBEA [38] are compared with the parameter setting summarized in Table 1. As can be seen from Figure 10, NSGA-II, SPEA2, IBEA and NSGA-III show close performance in our Braille block detection problem. Among them, NSGA-II has the highest performance. Also, as NSGA-II has fewer parameters that need to be adjusted compared to SPEA2 and NSGA-III, it is considered the most suitable off-the-shelf MOEA for solving our task in this paper. NSGA-III and SPEA2, which are based on Pareto domination, are considered to be able to perform a similar solution search as NSGA-II. Besides, Indicator-based IBEA has also shown competitive results. GDE3 fails in detecting some difficult test images and is more likely to be trapped by local optima in this task. Additionally, in Figure 11 we show the Pareto front approximation of NSGA-II obtained in the experiment. In the ideal case, as shown in the top image of Figure 11, we can observe a clear trade-off relationship between the two objective functions.

## 5. Conclusions

In this paper, we presented a method to detect Braille blocks under the framework of multi-objective optimization, which indicates that multi-objective optimization algorithms are potentially useful tools for solving real-world computer vision problems. Besides, we originally built a fully annotated dataset that contains five subcategories for validation. Experimental results show that the proposed method is effective in detecting Braille blocks from an egocentric viewpoint under real scenarios. As a limitation, our method tends to fail when either both of the features (geometric feature and color feature) are inadequate or one of the features are implausible. Nevertheless, in most cases, the algorithm driven by multi-object optimization can select a suitable solution from the solution space even one feature is inadequate due to illumination change, obstacle, deficiency or shadow. As future work, we aim at reducing the computational cost by enlarging the step for sampling test lines and patches for real-time applications, which can further contribute to the walking support for visually impaired people. 

## Figures and Tables

**Table 1:** Parameter setting in the experiment. Detail description is summarized in Table 2.

MOEA	POP	GEN	SR	PR	DIV	OS	DR	DS	NS	δ	η
NSGA-II [2]	200	50	1.0	0.25	-	-	-	-	-	-	-
SPEA2 [33]	200	50	1.0	0.25	-	200	-	-	-	-	-
IBEA [34]	200	50	1.0	0.25	-	-	-	-	-	-	-
GDE3 [35]	200	50	-	-	-	-	0.1	0.5	-	-	-
MOEA/D [36]	200	50	1.0	0.25	-	-	-	-	20	0.9	2
NSGA-III [37]	200	50	1.0	0.25	4	-	-	-	-	-	-
DBEA [38]	200	50	1.0	0.25	4	-	-	-	-	-	-

**Table 2:** Description of parameters shown in Table 1.

Par.	Description
POP	Population size.
GEN	Generation size.
SR	Crossover rate of the simulated binary crossover.
PR	Mutation rate of the polynomial mutation.
DIV	Number of divisions.
OS	Number of offspring generated per iteration.
DR	Crossover rate for differential evolution.
DS	Size of each step taken by differential evolution.
NS	Size of the neighborhood for mating.
δ	Probability of mating with an individual from the neighborhood versus the entire population.
η	Maximum number of spots in the population that an offspring can replace.

## Data Availability

Not applicable.
